# Changes in gene expression in healthcare workers during night shifts: implications for immune response and health risks

**DOI:** 10.1186/s40560-024-00769-5

**Published:** 2025-03-11

**Authors:** Ryota Nukiwa, Sayaka Oda, Hisatake Matsumoto, Mohamad Al Kadi, Shuhei Murao, Tsunehiro Matsubara, Shunichiro Nakao, Daisuke Okuzaki, Hiroshi Ogura, Jun Oda

**Affiliations:** 1 Department of Traumatology and Acute Critical Medicine, Osaka University Graduate School of Medicine, 2-15 Yamadaoka, Suita, Osaka 565-0871 Japan; 2 Department of Emergency and Critical Care Medicine, Hachinohe City Hospital, Aomori, Japan; 3 Laboratory for Human Immunology (Single Cell Genomics), WPI Immunology Frontier Research Center, Osaka University, Osaka, Japan

**Keywords:** Healthcare workers, Night shift, Health risk, Innate immune response, Whole-blood transcriptome analysis, Ingenuity Pathway Analysis (IPA), Major depressive disorder

## Abstract

**Background:**

Shift work is common in healthcare, especially in emergency and intensive care, to maintain the quality of patient care. Night shifts are linked to health risks such as cardiovascular disease, metabolic disorders, and poor mental health. It has been suggested that inflammatory responses due to the disruption of circadian rhythm may contribute to health risks, but the detailed mechanisms remain unclear. This study aimed to analyze changes in gene expression in whole blood of healthcare workers before and after a night shift and investigate the molecular pathogenesis of these changes and their impact on health.

**Methods:**

This was a single-center, prospective, observational study of four medical doctors working night shifts in the emergency department. Blood samples from the subjects were collected before and after the night shift, and RNA sequencing was performed to analyze changes in gene expression in whole blood. The data obtained were analyzed via Ingenuity Pathway Analysis (IPA) core analysis that included canonical pathway analysis, upstream regulator analysis, and functional network analysis. RNA bulk deconvolution was performed to estimate the relative abundance of immune cells. The IPA analysis match feature was also used to assess similarities of gene expression patterns with other diseases.

**Results:**

We identified 302 upregulated and 78 downregulated genes (*p* < 0.05, |log2-fold change|> 0.5) as genes whose expression changed after the night shift. Canonical pathway analysis revealed that Toll-like receptors and other innate immune response pathways were activated. Upstream regulator analysis and functional network analysis also consistently indicated a predicted activation of innate immune and inflammatory responses. RNA bulk deconvolution showed changes in the proportions of several immune cells. IPA analysis match indicated that gene expression patterns after night shifts were highly correlated with several diseases, including major depressive disorder, in terms of immune and inflammatory responses.

**Conclusion:**

The results revealed that innate immune and inflammatory responses are elicited after night shifts in healthcare workers and that gene expression patterns correlate with several diseases in terms of immune and inflammatory responses. These findings suggest that shift work may affect health risks through innate immune and inflammatory responses.

**Supplementary Information:**

The online version contains supplementary material available at 10.1186/s40560-024-00769-5.

## Introduction

In modern society, shift work has become an essential form of work in many industries. It is estimated that 20–30% of all workers in North America and Europe are engaged in shift work, including night shifts [1, 2]. Shift work is common in the medical field, especially in the emergency and intensive care fields, where doctors, nurses, and other healthcare workers need to be available to patients 24 h a day to maintain the quality of patient care. Moreover, many concerns have been raised about the impact of shift work on health.

In recent years, the effects of night shifts and irregular work on physical and mental health have been widely reported. Epidemiological studies have linked shift work to a wide range of increased risks, including cardiovascular disease, metabolic disorders such as diabetes, digestive disorders, sleep disorders, mood disorders, worsening mental health such as depression, and malignancies such as skin, breast, and prostate cancer [3, 4]. Changes in white blood cell counts such as monocyte, lymphocyte, and neutrophil counts and increased levels of inflammatory markers such as C-reactive protein (CRP) and interleukin (IL)6 have been reported due to night work [5–7]. Circadian rhythm disturbances due to shift work, including night shifts, may affect the inflammatory response and may be a health risk factor, but the detailed mechanisms are still not fully understood.

Recently, technological advances have made it possible to perform transcriptome analysis, which uses RNA sequencing to comprehensively measure changes in gene expression and reveal the clinical phenotype in molecular pathology. Furthermore, previously published RNA sequencing data are available in public databases, thus making it possible to compare one’s own research with existing studies. Such analysis is expected to elucidate both similarities and differences between different diseases, thereby contributing to a deeper understanding of disease mechanisms.

Few studies have conducted transcriptome analyses of whole blood of workers performing shift work. All of these previous studies have been conducted on healthy volunteers, and no studies have conducted whole-blood transcriptome analysis on actual healthcare workers or night shift workers.

The aim of this study was to comprehensively analyze changes in gene expression in whole blood of actual healthcare professionals before and after a night shift and to investigate the molecular pathologies that change after a night shift and their impact on health.

## Materials and methods

### Study design and participants

**Fig. 1:**
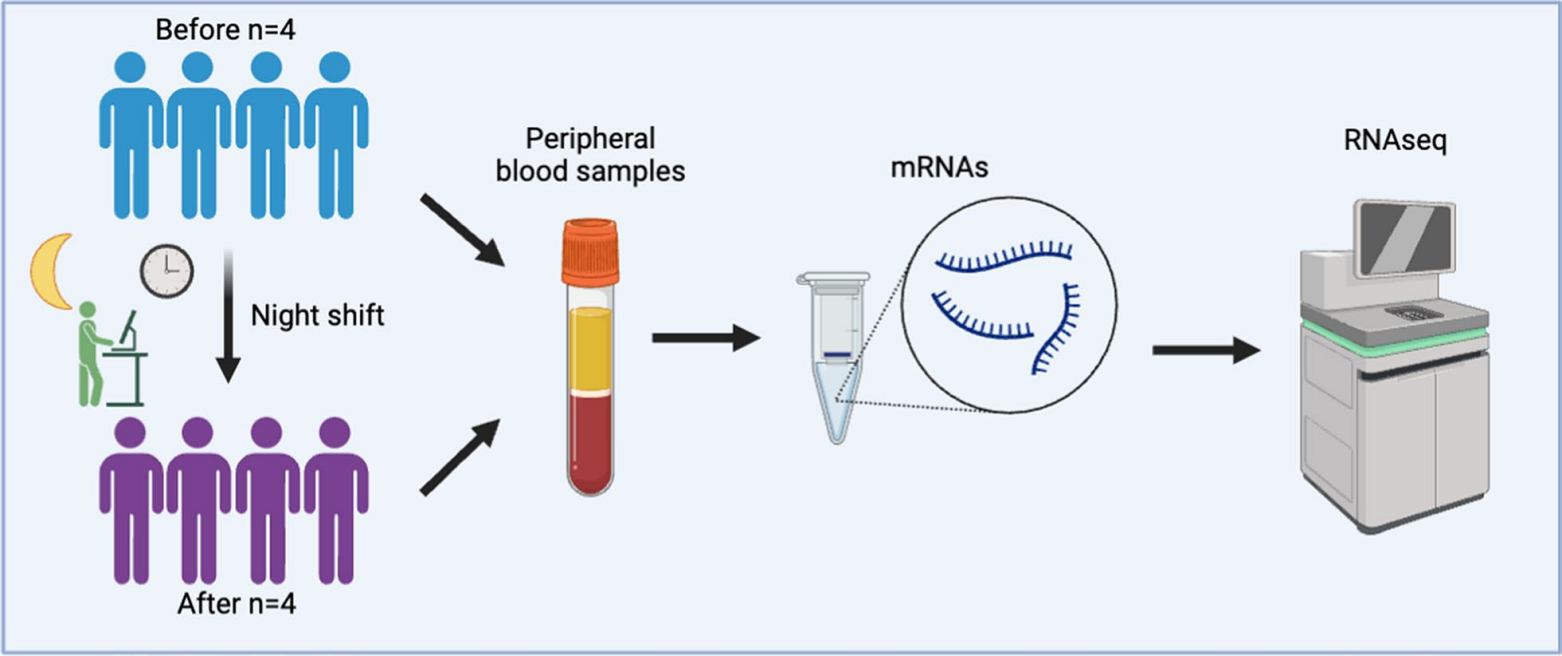
Workflow of our research

This single-center, prospective, observational study was conducted in September 2020 at the Department of Traumatology and Acute Critical Medicine, Osaka University Graduate School of Medicine. Blood samples were collected from four doctors working night shifts in emergency and intensive care departments at 10 am on the day of work and at 10 am the following day after work. Age, sex, body mass index, and comorbidities (e.g., hypertension, diabetes, hyperlipidemia) were investigated in all four subjects (Fig. 1).

This study was approved by the Institutional Review Board of Osaka University Hospital in June 2020 in accordance with the principles of the Declaration of Helsinki. (Approval no.: 907). Written informed consent for the study was obtained from the subjects at the time of blood sample collection.

### Emergency department setting

The emergency department handles approximately 1400 third-level emergency transports annually that deal with the most serious and life-threatening conditions. These include out-of-hospital cardiac arrest, trauma, sepsis, and cerebrovascular and cardiovascular emergencies. The department provides seamless care from initial treatment through intensive care management and has a 20-bed intensive care unit. The medical staff works on a two-shift system, with three doctors on duty during the night shifts who are responsible for both initial treatment and intensive care management.

### Assessment of fatigue

The visual analogue scale to evaluate fatigue severity (VAS-F) score was used to assess the subjects’ fatigue levels before and after the night shift. The VAS-F score is a relatively valid and reliable measure for subjective assessment of fatigue and energy levels [8]. It comprises 18 items, divided into a 13-item fatigue subscale and a 5-item energy subscale. The scale is self-reported, and each item describes the subjects’ current state on a 10-point scale between two extremes, such as “not at all tired” to “extremely tired”. Higher scores on the fatigue subscale indicate greater levels of fatigue, whereas lower scores on the energy subscale indicate lower levels of energy. Subjects were asked to complete the VAS-F at the time of sample collection before and after the night shift, and the mean scores on the fatigue and energy subscales before and after the night shift were evaluated. A paired *t*-test was used to evaluate the change in scores before and after the night shift for each subject.

### Sample preparation, RNA isolation, library preparation, and RNA sequencing

Peripheral blood from each subject was subjected to total RNA isolation via the PAXgene™ Blood RNA System (BD Bioscience, San Jose, CA, USA). Full-length cDNA (complementary DNA) was generated via a SMART-Seq HT Kit (Takara Bio, Mountain View, CA). Illumina libraries were prepared via a Nextera DNA Library Preparation Kit (Illumina) according to the SMARTer kit instructions. The DNA library was then converted into a library for DNBSEQ via an MGIEasy Universal Library Conversion Kit (App-A). Sequencing was performed on the DNBSEQ-G400RS platform (MGI Tech Co., Ltd., Shenzhen, China) in 2 × 100 bp paired-end mode.

### RNA sequencing analysis

RNA sequencing analysis was performed as previously described [9]. The sequenced reads were mapped to the human reference genome sequence (hg19) via TopHat (version 2.1.1) in conjunction with Bowtie2 (version 2.2.8) and SAMtools (version 0.1.18). Raw read counts of gene-level expression for each gene‒sample combination were calculated via featureCounts in the subread-2.0.0 package.

### Statistical analysis of mRNA

Analyses were performed as previously described, with some modifications [9]. Integrated Differential Expression and Pathway Analysis, version 2.01 (iDEP.2.01, http://bioinformatics.sdstate.edu/idep/) [10] was used to normalize the raw mRNA (messenger RNA) count data. The raw counts were converted to CPM (counts per million) on iDEP.2.01, with default settings (min. CPM 0.5, n libraries1), and genes with low expression were filtered. Log2 normalization was also performed via the EdgeR algorithm, with the pseudo count set to the default setting of 4. To compare gene expression between subjects before and after the night shift, principal component analysis was performed: the limma-voom algorithm [11] was used to analyze expression variation between subjects before and after the night shift. Paired tests were also performed for the before-shift and after-shift samples of the same subject as the corresponding paired samples. |Log2-fold change|> 0.5 and p < 0.05 were used to define differentially expressed genes (DEGs), and MA plots were drawn to visualize significant changes in the expression list.

### Analysis by Ingenuity Pathway Analysis core analysis

Ingenuity Pathway Analysis (IPA, 2024 summer) (QIAGEN, https://digitalinsights.qiagen.com/products-overview/discovery-insights-portfolio/analysis-and-visualization/qiagen-ipa/) was used to perform IPA core analysis to assess the functional characteristics of mRNA expression, upstream regulators of mRNAs, and functional networks by expression variation genes. Canonical pathway analysis, upstream regulator analysis, and regulator effect analysis were conducted as IPA core analyses [12]. The p values were calculated via the Benjamini–Hochberg method with multiple comparison test correction [13]. The analysis uses z scores to predict the activation status of canonical pathways and upstream regulators based on mRNA expression patterns: higher z scores indicate greater activation, whereas lower z scores suggest greater inhibition. In the present study, |z score|≥ 2, *p* < 0.05, was considered to indicate significant activation or inhibition. Regulator effect analysis was performed to analyze the functional network, which theoretically predicts downstream disease/function caused by DEGs and upstream regulators predicted from the DEGs. Consistency scores were calculated as a measure of how consistent the upstream-to-downstream functional network was with the published literature.

### RNA bulk deconvolution analysis by CIBERSORTx

To estimate the proportions of immune cells from the RNA sequencing data, a deconvolution analysis was performed using the web tool CIBERSORTx (https://cibersortx.stanford.edu/), a machine learning algorithm [14]. RNA sequencing data were converted into a gene expression profile matrix that was input into CIBERSORTx. We ran “Impute Cell Fractions” with default settings using the LM22 signature matrix as the reference. LM22 is a leukocyte gene signature matrix provided by CIBERSORTx that contains 547 genes identifying 22 different human immune cell types [15]. The output of CIBERSORTx lists the relative abundance of 22 immune cell subtypes for each sample. Among the immune cell subtypes, changes in the proportions of neutrophils, lymphocytes, and monocytes were analyzed before and after the night shift. Lymphocytes were calculated by summing the percentages of naive B cells, memory B cells, plasma cells, CD8 T cells, naive CD4 T cells, resting memory CD4 T cells, activated memory CD4 T cells, follicular helper T cells, regulatory T cells, γδ T cells, resting natural killer (NK) cells, and activated NK cells. Paired *t*-tests were performed to analyze the percentage changes in estimated immune cells proportions before and after the night shift.

### Analysis by IPA analysis match

IPA analysis match was performed to compare the similarity between the IPA-analyzed core analysis results and the IPA-pre-analyzed core analysis results from expression data derived from public databases. To narrow down the core analysis results related to disease, the results were filtered by “human disease”, “peripheral blood”, and “disease vs. normal”, and a similarity analysis was performed via the dataset match metric. This metric compares the gene sets analyzed via IPA with pre-analyzed gene sets in the IPA collection to identify common genes, pathways, diseases, and functions. The metric quantitatively assesses the degree of similarity of the compared gene sets via z scores and p values: a higher or lower z score indicates a positive or negative correlation, respectively, and a lower p value indicates a significant gene match. The top 20 diseases with similar expression levels obtained from IPA analysis match were selected after excluding duplicate diseases. A heatmap was created for the selected diseases on the basis of the z scores of the terms for each of the three analytical metrics: canonical pathway, upstream regulators, and downstream effects. Additionally, hierarchical clustering was performed for each disease via the *z* scores of the respective terms for these three analytical metrics.

### Validation of the results obtained by IPA

To validate the results of the canonical pathway analysis by IPA, pathway analysis (Kyoto Encyclopedia of Genes and Genomes [KEGG], Reactome) and Gene Ontology (GO) analyses (GO biological process [GOBP], GO molecular function [GOMF]) of the DEGs were performed via the web tool Enrichr (https://maayanlab.cloud/Enrichr/) [16–20]. To validate the results of IPA analysis match, gene set enrichment analysis software (GSEA, v4.3.3) was used [21]. Correlations between the gene sets of the abovementioned diseases obtained via IPA analysis match and the DEGs obtained in the present study were evaluated. DEGs with fold change data were converted into pre-ranked lists and input into GSEA software to calculate the normalized enrichment score, nominal p value, and false discovery rate, which indicate the level of correlation.

## Results

### Subject characteristics

Four medical doctors participated as the study subjects. Their median age was 37 (interquartile range: 34.5–38.3) years, and their median body mass index was 22.4 (interquartile range: 21.8–23.3) kg/m^2^. None of the subjects experienced any complications (Table 1).

**Table 1:** Baseline characteristics of the participants

Characteristic	Subjects (*n* = 4)
Age, years	37 (34.5–38.3)
Male, *n* (%)	4 (100%)
BMI, kg/m^2^, median (IQR)	22.4 (21.8–23.3)
Comorbidities, *n* (%)	
Diabetes	0 (0)
Hypertension	0 (0)
Hyperlipidemia	0 (0)
Chronic lung disease	0 (0)
Chronic kidney disease	0 (0)
Immunocompromised condition	0 (0)

### Assessment of fatigue via VAS-F score

The degree of fatigue in the subjects before and after the night shift was evaluated using the VAS-F score (Table 2). The fatigue subscale was significantly higher after the night shift than before (mean score ± SD before vs. after: 1.2 ± 1.4 vs. 7.4 ± 1.1, *p* = 0.002). The energy subscale tended to be lower after the night shift than before (mean score ± SD before vs. after: 7.1 ± 1.1 vs. 3.3 ± 2.1, *p* = 0.069). These results indicated that the subjects felt fatigued after the night shift.

**Table 2:** Differences in the degree of fatigue using the VAS-F score before and after the night shift

Scale	Before	After	*p* value
VAS-F			
Fatigue	1.2 (1.4)	7.4 (1.1)	0.002
Energy	7.1 (1.1)	3.3 (2.1)	0.069

Data are presented as the mean (SD) of the VAS-F score

*p* values are analyzed using the paired *t*-test

*VAS-F*, visual analogue scale to evaluate fatigue severity

### Comparison of gene expression

**Fig. 2:**
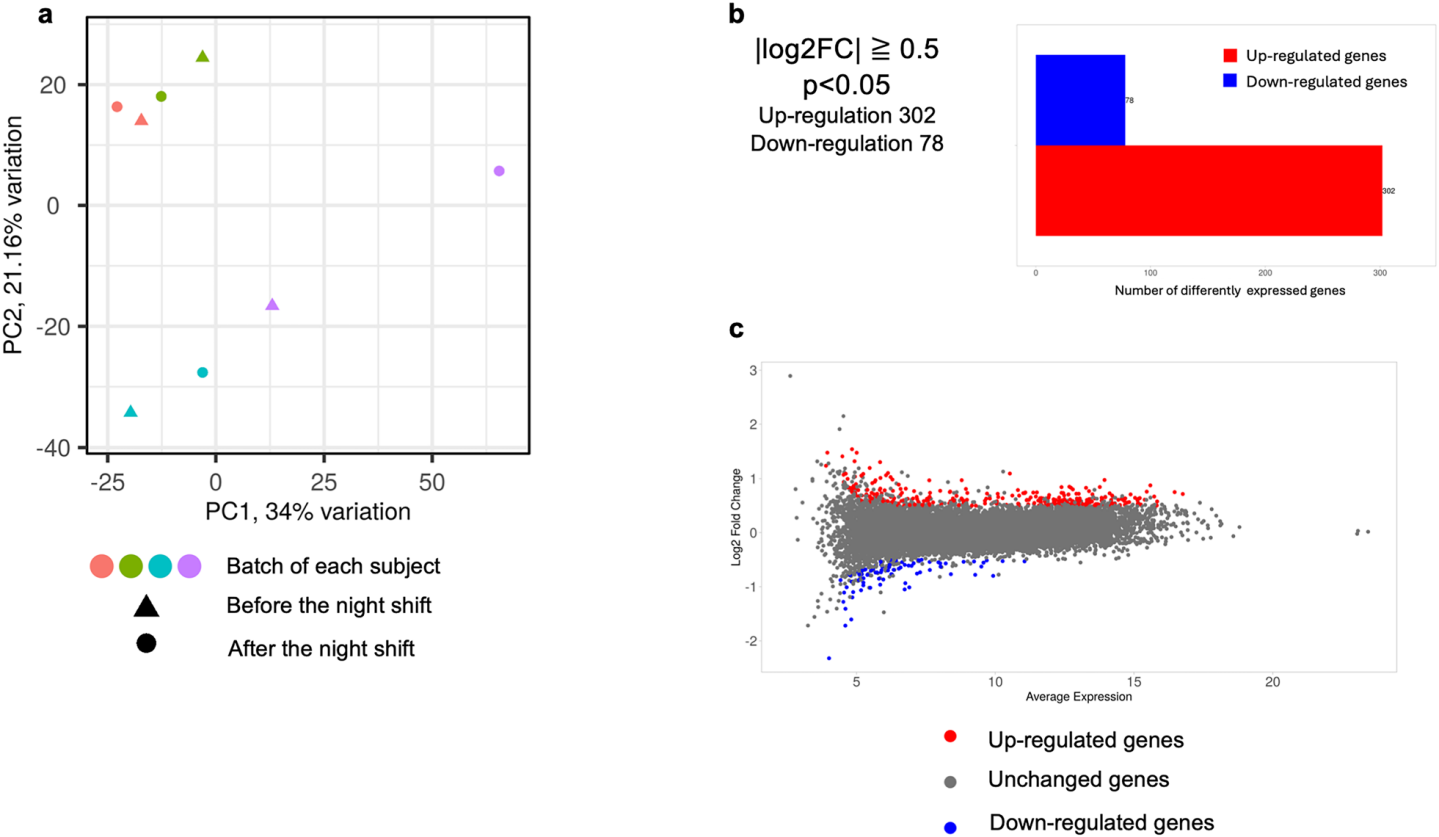
Characteristics of changes in mRNA expression before and after the night shift. **a** Principal component analysis comparing mRNA expression before and after the night shift. The same color indicates the same subject, with triangles indicating before the night shift, and circles indicating after the night shift. **b** Gene expression changes occurring after the night shift (p < 0.05, |log2-fold change|> 0.5). The mRNA expression of 302 genes increased, and that of 78 genes decreased after the night shift. **c** The colored dots indicate genes with variable expression (p < 0.05, |log2-fold change|> 0.5). The red dots indicate upregulated genes, and the blue dots indicate downregulated genes

Principal component analysis revealed no distinct or obvious differences in mRNA expression patterns before and after the night shift for each subject (Fig. 2a). Use of the limma-voom algorithm for paired analysis showed that the expression of 302 genes was upregulated, and that of 78 genes was downregulated after the night shift (*p* < 0.05 and |log2-fold change|≥ 0.5) (Fig. 2b, c).

### Canonical pathway analysis

**Fig. 3:**
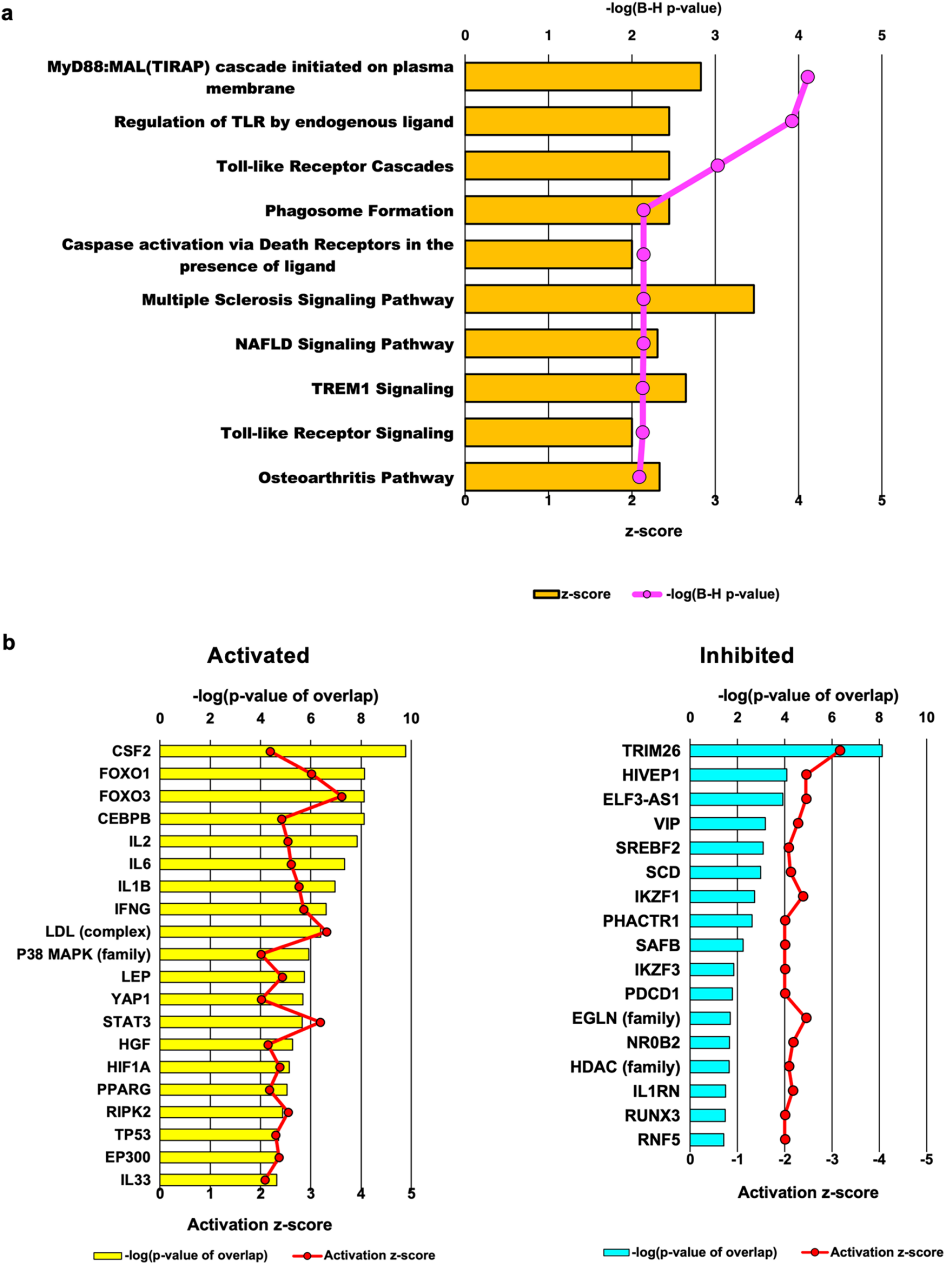
Canonical pathway analysis and upstream regulator analysis. **a** Top 10 canonical signaling pathways that fluctuated after the night shift were identified via Ingenuity Pathway Analysis (IPA). The bars represent z scores, and the line graphs represent logarithmically adjusted p values associated with each pathway. **b** Upstream regulators predicted after the night shift identified via IPA. The left graph shows the top 20 upstream regulators predicted to be activated after the night shift. The right graph shows the top 17 upstream regulators predicted to be inhibited after the night shift. The bars indicate the logarithm of the overlap of the p values of the transcriptional regulators, and the line graphs indicate the z values

The results of RNA sequencing were submitted to IPA, and canonical pathway analysis was performed. Canonical signaling pathways that were activated or inhibited after the night shift were identified and listed. Canonical pathway analysis predicted 16 pathways to be activated and one pathway to be inhibited (|z score|≥ 2; *p* value of overlap < 0.05). The top 10 pathways are shown in Fig. 3a. The myeloid differentiation primary response 88 (MyD88):MyD88 adapter like protein (MAL) (Toll-interleukin 1 receptor domain containing adaptor protein [TIRAP]) cascade initiated on the plasma membrane had the lowest p value and was the most activated (adjusted *p* value = 7.8 × 10^–5^; *z* score = 2.828). Other activated pathways included the regulation of Toll-like receptor (TLR) by endogenous ligand, TLR cascades, triggering receptor expressed on myeloid cells (TREM)1 signaling, and TLR signaling, with a dominant presence of pathways related to the innate immune response, particularly those involving TLRs.

Pathway analysis (Reactome, KEGG) and GO analysis (GOBP, GOMF) were performed via the web tool Enrichr to validate the results of IPA (Supplemental Fig. S1). Enrichr analysis revealed terms similar to those observed in the IPA canonical pathway analysis, such as the regulation of TLRs by endogenous ligands and the MyD88:MAL (TIRAP) cascade initiated on the plasma membrane, supporting the results of the canonical pathway analysis.

### Upstream regulator analysis

Upstream regulator analysis predicted 84 activated and 17 inhibited potential upstream regulators whose expression varied after the night shift (*p* value of overlap < 0.05). These top-ranked upstream regulators are shown in Fig. 3b. Among the activated upstream regulators, inflammatory cytokines such as colony stimulating factor (CSF)2, IL2, IL6, IL1b, and interferon-γ (IFNG); inflammation-related transcription factors such as signal transfer and activator of transcription (STAT)3 and hypoxia inducible factor (HIF)1; and inflammation-related signaling factors such as p38 mitogen activated protein kinase (p38 MAPK) (family) were identified. Transcription factors such as forkhead box O (FOXO)1 and FOXO3 were also detected. Among the inhibited upstream regulators, the ubiquitin ligase tripartite motif containing (TRIM)26 was found.

### Functional network analysis

**Fig. 4:**
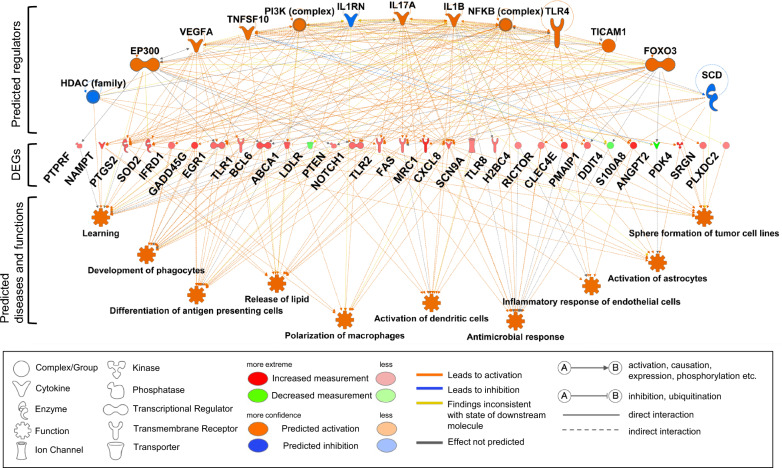
Predicted functional networks observed after the night shift. The consistency score of the top network is shown. Upstream regulators (top) are predicted to be associated with disease and function (bottom) via gene sets consisting of differentially expressed genes (DEGs) (middle) before and after the night shift. Red indicates genes that are actually upregulated, green genes that are downregulated, orange upstream molecules/downstream factors that are predicted to be activated, and blue upstream molecules/downstream factors that are predicted to be suppressed. The meaning of each shape, line and color is also shown

Next, we performed regulator effect analysis and predicted functional networks based on the consistency score. The top five networks as ranked by consistency score are listed in Supplemental Table S1, and the top-ranked network is shown in Fig. 4. The top-ranked network included 13 upstream regulators, 29 DEGs, and 10 diseases/functions. The downstream diseases/functions included terms such as development of phagocytes, differentiation of antigen-presenting cells, polarization of macrophages, activation of dendritic cells, antimicrobial response, and inflammatory response of endothelial cells. Many of these downstream functions were related to innate immune responses, including the activation of phagocytes and antigen-presenting cells, and the inflammatory response in epithelial cells. The other functional networks listed in the abovementioned table also showed a similar trend.

### RNA bulk deconvolution analysis via CIBERSORTx

The RNA sequencing data were deconvolved using CIBERSORTx to analyze the changes in immune cells before and after the night shift for each subject. The estimated proportions of immune cells in each subject’s blood are shown as a stacked bar graph in Supplemental Fig. S2a. Several immune cells showed changes in proportions before and after the night shift. Changes in the percentages of neutrophils, lymphocytes, and monocytes in each subject before and after the night shift are also shown in Supplemental Fig. S2a. The percentages of neutrophils tended to increase, whereas those of lymphocytes and monocytes tended to decrease, but the differences were not statistically significant.

### Analysis by IPA analysis match

**Fig. 5:**
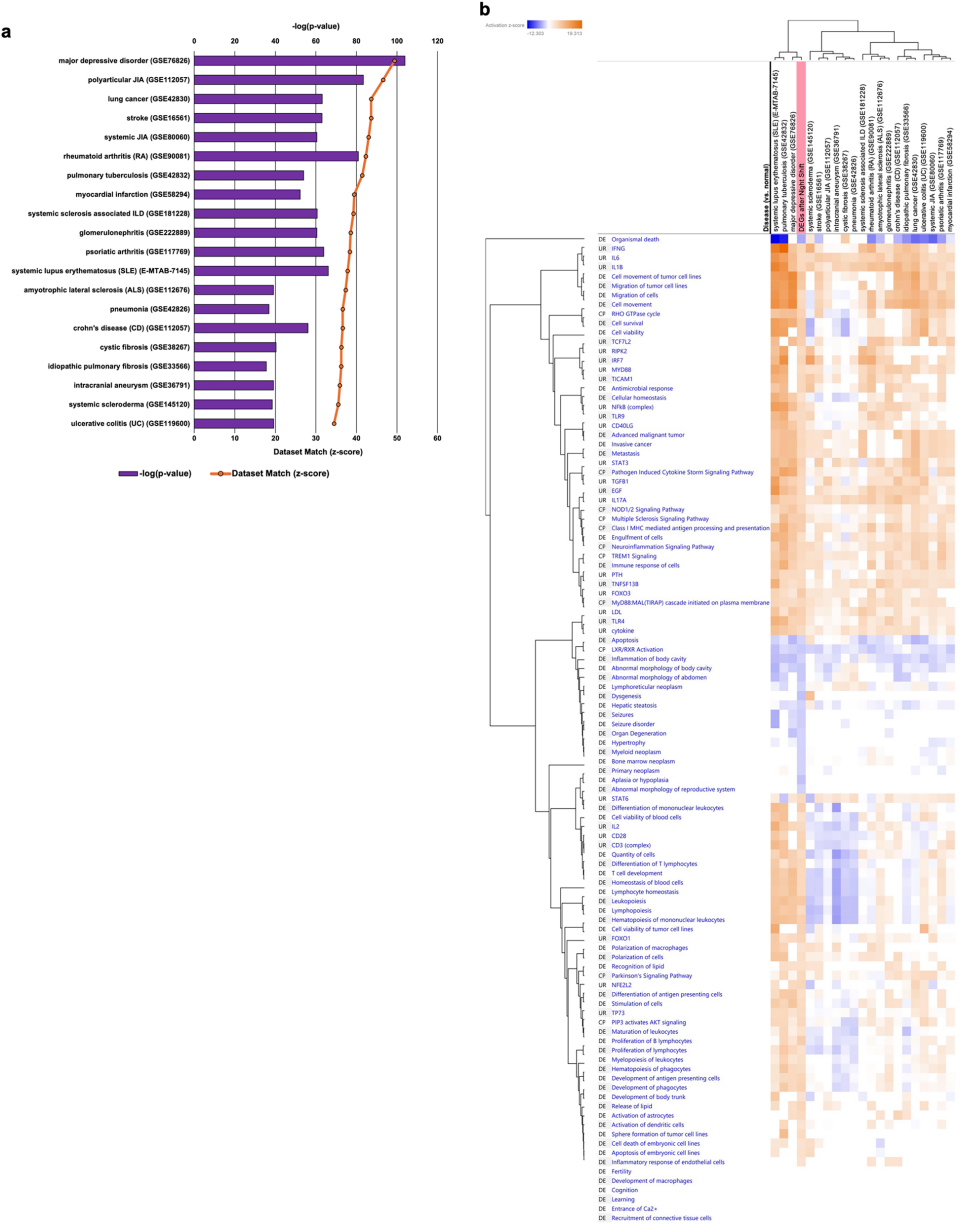
Other diseases showing similar expression patterns to those of DEGs after the night shift. The data were analyzed via analysis match of Ingenuity Pathway Analysis via dataset match metrics. **a** The top 20 z scores for the strength of correlation are shown. Bars indicate p values, and line graphs indicate z scores. **b** Heatmaps and hierarchical clustering of three analytical metrics—canonical pathway, upstream regulators, and downstream effects—in the top 20 other diseases showing similar expression patterns to those of DEGs after the night shift. The vertical columns indicate the terms for each canonical pathway, upstream regulators, and downstream effects. The horizontal column lists 20 diseases and their DEGs before and after the night shift. The DEGs before and after the night shift are highlighted in pink. The heatmap shows the z score of each disease for each of the three analysis indices for each term, with high z scores in orange and low z scores in blue. *DEGs* differentially expressed genes

The top 20 diseases with the highest z scores, indicating the strength of correlation as determined by IPA analysis match, are shown in the figure, with overlapping diseases excluded (Fig. 5a, Supplemental Table S2). All diseases showed statistically significant correlations. Interestingly, major depressive disorder was most strongly correlated with gene expression changes before and after the night shift (*p* value = 1.1 × 10^–104^; *z* score = 49.437). Other observed diseases included autoimmune diseases such as polyarticular or systemic juvenile idiopathic arthritis, rheumatoid arthritis, and systemic lupus erythematosus, which are highly prevalent. Additional diseases included malignant tumors such as lung cancer; ischemic diseases such as stroke and myocardial infarction; and infectious diseases such as pulmonary tuberculosis and pneumonia. Heatmap and hierarchical clustering analyses were performed for each disease based on the z scores of the three analytical metrics: canonical pathways, upstream regulators, and downstream effects (Fig. 5b). Hierarchical clustering also indicated that the changes in gene expression before and after the night shift and the gene set of major depressive disorder were the closest, with similar z scores on the heatmap, suggesting a high degree of correlation.

To validate the results of the IPA analysis match, the top six diseases with high similarity were examined for gene expression correlations via GSEA software. All diseases were significantly highly correlated (Supplemental Fig. S3).

## Discussion

In this study, whole-blood RNA sequencing was performed before and after the night shift in four physicians working the night shift to investigate changes in gene expression. Principal component analysis showed differences in mRNA expression patterns between the subjects. As paired tests were conducted on the same subjects before and after the night shift, and the downstream analysis was performed using only statistically significant DEGs, the influence of inter-subject differences was considered to be minimal. IPA core analysis was conducted, and canonical pathway analysis, upstream regulator analysis, and functional network analysis consistently showed that innate immune responses and inflammatory reactions were activated after the night shift. RNA bulk deconvolution also showed changes in the proportions of several immune cells. In addition, IPA analysis match revealed that gene expression patterns after the night shifts were highly correlated with several diseases, including major depressive disorder.

### Relationship between shift work and inflammatory response in existing studies

Previous studies have reported increases in inflammatory markers such as CRP and IL6 after night shift work [5–7]. Night shifts have also been associated with changes in white blood cell counts, such as monocyte, lymphocyte, and neutrophil counts [22, 23], and these changes have been reported to be more pronounced in workers who work more frequent and consecutive night shifts [24].

Atwater et al. reported that in addition to elevated inflammatory cytokines and increased leukocytes, the level of lipopolysaccharide-binding protein was also elevated after night shifts. They concluded that shift work, sleep deprivation, stress, and circadian rhythm disturbances may increase intestinal permeability and lipopolysaccharide-binding protein by transferring lipopolysaccharide into the blood, thus contributing to an enhanced innate immune response and inflammatory response [25].

Several studies have used transcriptome analysis of peripheral blood and peripheral blood mononuclear cells in experiments in which healthy volunteers were placed in a simulated night shift environment to analyze sleep deprivation, such as that caused by shift work and jet lag. Disruption of circadian rhythms, such as through sleep deprivation and shift work, alters the expression rhythm of clock-related genes, reducing their expression amplitude and altering the expression of genes involved in innate immune responses and inflammation, such as MAL, TREM1, IL6, and STAT3 [26–28].

### Results of the IPA core analysis and RNA bulk deconvolution

In this study, the most activated pathway in the canonical pathway analysis was the MyD88:MAL (TIRAP) cascade initiated on the plasma membrane. MyD88 is an adaptor protein that signals downstream of pattern-recognition receptor TLRs expressed on macrophages and dendritic cells and is involved in the initiation of TLR-mediated innate immune responses. Other innate immune response-related pathways, such as TLR-related pathways, are also activated.

Upstream regulator analysis also suggested that the potentially activated upstream regulators included inflammatory cytokines such as CSF2, IL2, IL6, IL1b, and IFNG; inflammation-related transcription factors such as STAT3 and HIF-1; and inflammation-related signaling factors such as p38 MAPK, indicating that upstream regulators related to the innate immune response and inflammatory response factors were activated.

Functional network analysis predicted the differentiation, maturation, and activation of phagocytes such as macrophages, dendritic cells, and antigen-presenting cells, as well as immune responses in epithelial cells, and predicted functional changes centered on innate immune responses.

In summary, IPA core analysis that included canonical pathway analysis, upstream regulator analysis, and functional network analysis consistently indicated that inflammatory responses centered on innate immune responses are elicited after the night shift.

In addition, we performed RNA bulk deconvolution to estimate the proportions of immune cells from the gene expression results and found that the proportions of immune cells changed before and after the night shift. The results of IPA core analysis and RNA bulk deconvolution were consistent with those of previous studies [22–24, 26–28].

These results showed that the changes in gene expression related to innate immune and inflammatory responses and those in immune cell proportions were observed after the night shift in actual healthcare workers working the night shift.

### Relationships between shift work and other diseases according to existing studies

With respect to shift work and health risks, epidemiological studies have reported an association between shift work and metabolic diseases such as type 2 diabetes, coronary artery disease, stroke, certain types of cancer, mental disorders such as depression, and autoimmune diseases [3, 4].

A meta-analysis assessing the impact of shift work on mental health revealed that shift work was associated with a 1.28-fold increased risk of mental health deterioration in general and a 1.33-fold increased risk of depression in particular. The combined effects of circadian rhythm disturbances, sleep deprivation, and other factors are considered to be the cause [29].

Approximately one-third of depressed patients have elevated inflammatory markers even in the absence of medical illness, and patients with inflammatory diseases and those receiving cytokine therapy, such as interferon, are more likely to develop depression [30, 31]. Inflammatory mediators affect neurotransmission and neuroendocrine function and may contribute to the pathophysiology of depression [32]. These findings suggest that depression and inflammation may bidirectionally influence each other.

### Results of the IPA analysis match

In this study, IPA analysis match was used to identify diseases with similar gene expression patterns to those observed after night shifts, and interestingly, major depressive disorder had the highest correlation. Other diseases showing correlations were autoimmune diseases, malignant tumors, ischemic diseases, and infectious diseases. Heatmap and hierarchical clustering analyses were performed for each disease based on z scores of the three analytical metrics. Hierarchical clustering analysis indicated that changes in gene expression after the night shift and major depressive disorder were the most closely related. The heatmaps showed similarities in the colors of the z scores related to the innate immune response and the inflammatory response. These findings suggest that gene expression changes after a night shift are correlated with major depressive disorder in terms of the innate immune response and inflammatory response.

Previous studies have shown that shift work increases the risk of depression and that depression is related to the inflammatory response. In terms of the gene expression variation in the present study, the results suggest that shift work may increase the expression of genes related to innate immune responses and inflammation and potentially increase the risk of depression.

### Implications of the study findings

This study examining changes in gene expression in the blood of healthcare workers before and after the night shift work revealed that the innate immune response and inflammatory response are elicited after night shift work. In addition, gene expression changes before and after the night shift were correlated with gene expression changes in major depressive disorder and several other diseases, suggesting that shift work may affect health risks through innate immune and inflammatory responses. The findings of this study may provide a basis for future research on shift work and health risks, including major depressive disorder.

### Strengths and limitations

The present study has several strengths. Relatively few studies have conducted transcriptome analyses of whole blood from shift workers. Further, all previous reports were conducted on healthy volunteers in simulated night shift or sleep deprivation environments. To the best of our knowledge, this is the first report of whole-blood transcriptome analysis and gene expression analysis conducted in actual healthcare workers working the night shift and is also the first study to analyze similarities and correlations with other diseases via IPA analysis match.

The present study also has several limitations. First, this study was exploratory in nature and included a small sample size. The small sample size and homogeneity of the participants, who were all doctors from a single institution with similar backgrounds (e.g., gender, age, race, and job descriptions), may have introduced selection bias, which potentially limits the generalizability of the findings to broader populations. The insufficient sample size also reduced the statistical power, which could lead to overinterpretation or underestimation of the results. Second, the VAS-F score used in this study to evaluate the subjects’ level of fatigue is a self-reported type of subjective evaluation, and the criteria for evaluating the degree of fatigue may differ from subject to subject. However, this study evaluated the change in each subject’s level of fatigue before and after the night shift, and the effect of differences in the way each subject felt fatigued was considered minimal. Third, this study did not assess working conditions (e.g., stress, busyness, breaks, sleeping time), so other factors may have influenced the results. Fourth, only changes in gene expression were assessed one at a time before and after the night shift, and owing to the short observation period, the long-term effects are unclear. Fifth, the analysis methods are limited. RNA sequencing and IPA were the main methods used in this study, but a more comprehensive understanding could be achieved by analyzing blood markers and combining other analysis methods. Finally, although this study described a possible association with several diseases, including major depressive disorder, on the basis of similarities in gene expression changes, no other validation has been conducted, and the actual associations are unknown. Therefore, larger, long-term studies, integrated approaches using multiple analytical methods, and intervention studies are desirable to further validate these results.

## Conclusion

We examined gene expression changes in whole blood before and after the night shift in healthcare workers and showed that innate immune and inflammatory responses are elicited after the night shift. The present findings suggest that shift work may affect health risks through innate immune and inflammatory responses. Further studies are warranted to confirm these findings.

## Supplementary Information

Additional file 1. **Supplemental Fig. S1.** Validation of canonical pathway analysis by Enrichr. The top 10 Reactome, KEGG, GOBP, and GOMF terms significantly enriched in DEGs. The bars represent logarithmically adjusted p values associated with each term. *DEGs* differentially expressed genes, *KEGG* Kyoto Encyclopedia of Genes and Genomes, *GOBP* Gene Ontology biological process, *GOMF* Gene Ontology molecular function. **Supplemental Fig. S2.** Results of estimation of the relative abundance of immune cells in blood by RNA bulk deconvolution using CIBERSORTx. **a** Stacked bar graphs show the estimated relative abundance of 22 immune cells determined before and after the night shift for each subject. Each color represents the immune cell indicated in the legend. **b** Changes in the percentages of neutrophils, lymphocytes, and monocytes in each subject before and after the night shift. Statistical tests were performed using the paired *t*-test, with p < 0.05 considered a significant difference. *NK* natural killer, *CD* cluster of differentiation, *ns* not significant. **Supplemental Fig. S3.** Validation of analysis match of Ingenuity Pathway Analysis via gene set enrichment analysis software. Enrichment plots of the top six diseases with high similarity are shown. Genes are ranked along the x-axis by the fold change data of DEGs in each disease. A vertical line along the x-axis indicates the genes present in the DEGs after the night shift. The green line represents the enrichment score at that position on the ranked gene list. The normalized enrichment score (NES), nominal p value, and false discovery rate (FDR) are indicated. *DEGs* differentially expressed genes, *JIA* juvenile idiopathic arthritis.

Additional file 2.

## Data Availability

The data presented in this study have been submitted to the National Center for Biotechnology Information (NCBI) Gene Expression Omnibus (accession number: GSE282051) for future access.

## Abbreviations

CD: Cluster of differentiation
cDNA: Complementary DNA
CPM: Counts per million
CRP: C-reactive protein
CSF: Colony stimulating factor
DEGs: Differentially expressed genes
FOXO: Forkhead box O
GO: Gene Ontology
GOBP: GO biological process
GOMF: GO molecular function
GSEA: Gene set enrichment analysis
HIF: Hypoxia inducible factor
iDEP: Integrated differential expression and pathway analysis
IFNG: Interferon-γ
IL: Interleukin
IPA: Ingenuity Pathway Analysis
KEGG: Kyoto Encyclopedia of Genes and Genomes
MAL: MyD88 adapter like protein
mRNA: Messenger RNA
MyD88: Myeloid differentiation primary response 88
NK: Natural killer
p38 MAPK: P38 mitogen activated protein kinase
STAT: Signal transfer and activator of transcription
TIRAP: Toll-interleukin 1 receptor domain containing adaptor protein
TLR: Toll-like receptor
TRIM: Tripartite motif containing
VAS-F: Visual analogue scale to evaluate fatigue severity
