# Supplementary material for: Changes in gene expression in healthcare workers during night shifts: implications for immune response and health risks
Source: J Intensive Care. 2025 Mar 11;13:14. doi: 10.1186/s40560-024-00769-5 (PMC11895378; doi:10.1186/s40560-024-00769-5)
Supplement: Supplementary file 1 — Additional file 1. Supplemental Fig. S1. Validation of canonical pathway analysis by Enrichr. The top 10 Reactome, KEGG, GOBP, and GOMF terms significantly enriched in DEGs. The bars represent logarithmically adjusted p values associated with each term. DEGs differentially expressed genes, KEGG Kyoto Encyclopedia of Genes and Genomes, GOBP Gene Ontology biological process, GOMF Gene Ontology molecular function. Supplemental Fig. S2. Results of estimation of the relative abundance of immune cells in blood by RNA bulk deconvolution using CIBERSORTx. a Stacked bar graphs show the estimated relative abundance of 22 immune cells determined before and after the night shift for each subject. Each color represents the immune cell indicated in the legend. b Changes in the percentages of neutrophils, lymphocytes, and monocytes in each subject before and after the night shift. Statistical tests were performed using the paired t-test, with p < 0.05 considered a significant difference. NK natural killer, CD cluster of differentiation, ns not significant. Supplemental Fig. S3. Validation of analysis match of Ingenuity Pathway Analysis via gene set enrichment analysis software. Enrichment plots of the top six diseases with high similarity are shown. Genes are ranked along the x-axis by the fold change data of DEGs in each disease. A vertical line along the x-axis indicates the genes present in the DEGs after the night shift. The green line represents the enrichment score at that position on the ranked gene list. The normalized enrichment score (NES), nominal p value, and false discovery rate (FDR) are indicated. DEGs differentially expressed genes, JIA juvenile idiopathic arthritis. [file 40560_2024_769_MOESM1_ESM.pptx]

## Slide 1
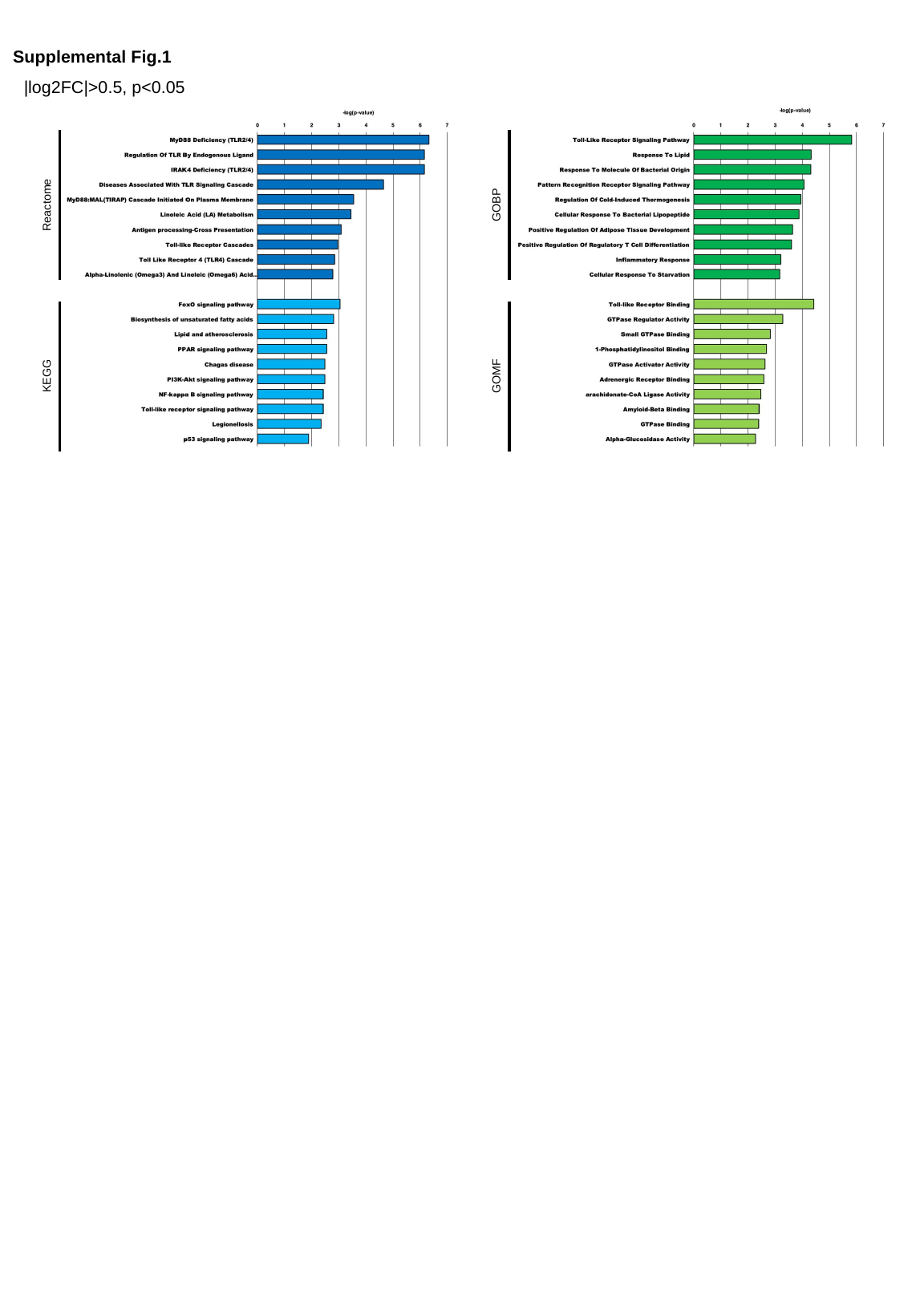

Supplemental Fig.1
|log2FC|>0.5, p<0.05
Reactome
GOBP
KEGG
GOMF

## Slide 2
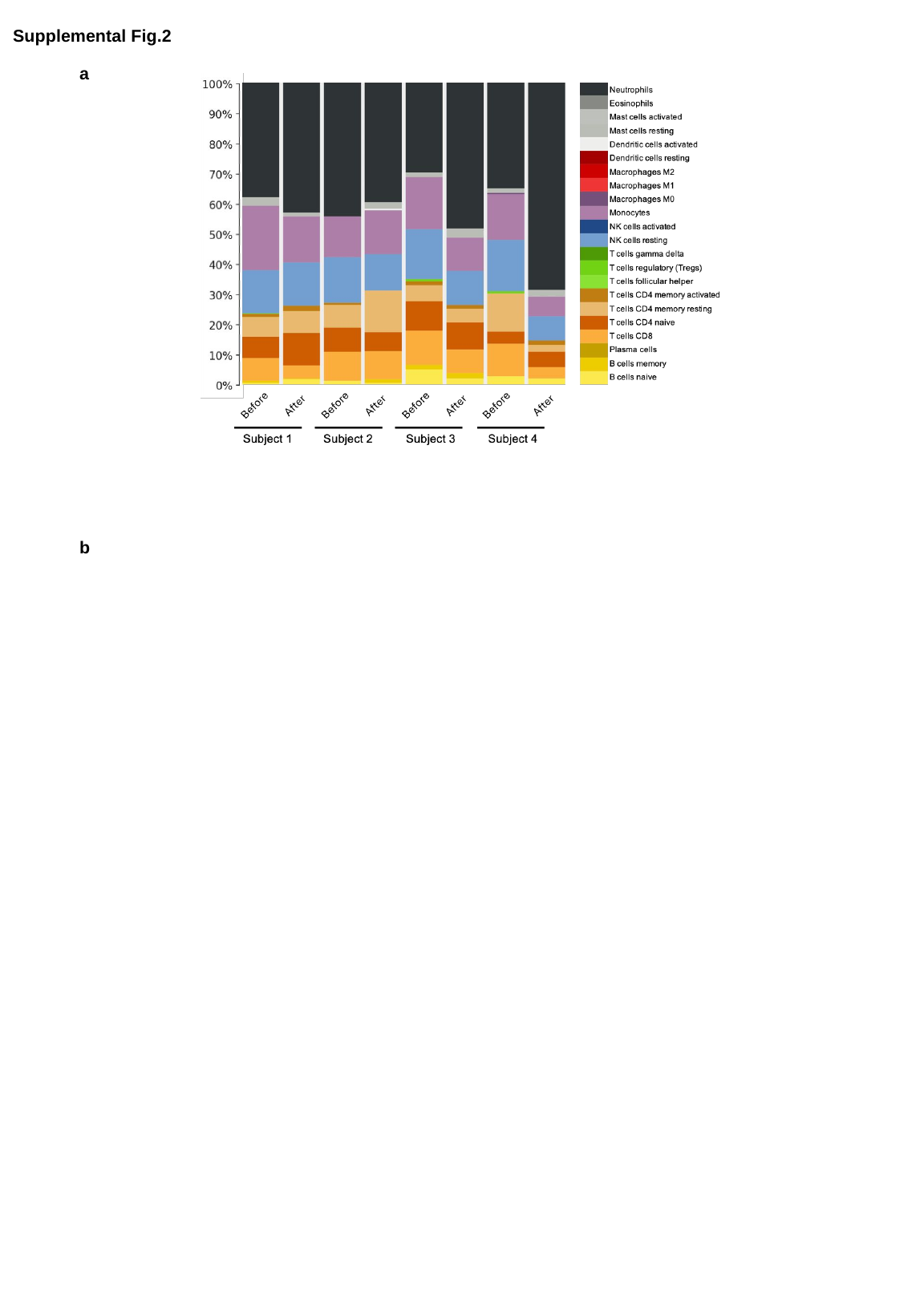

Supplemental Fig.2
a
b

## Slide 3
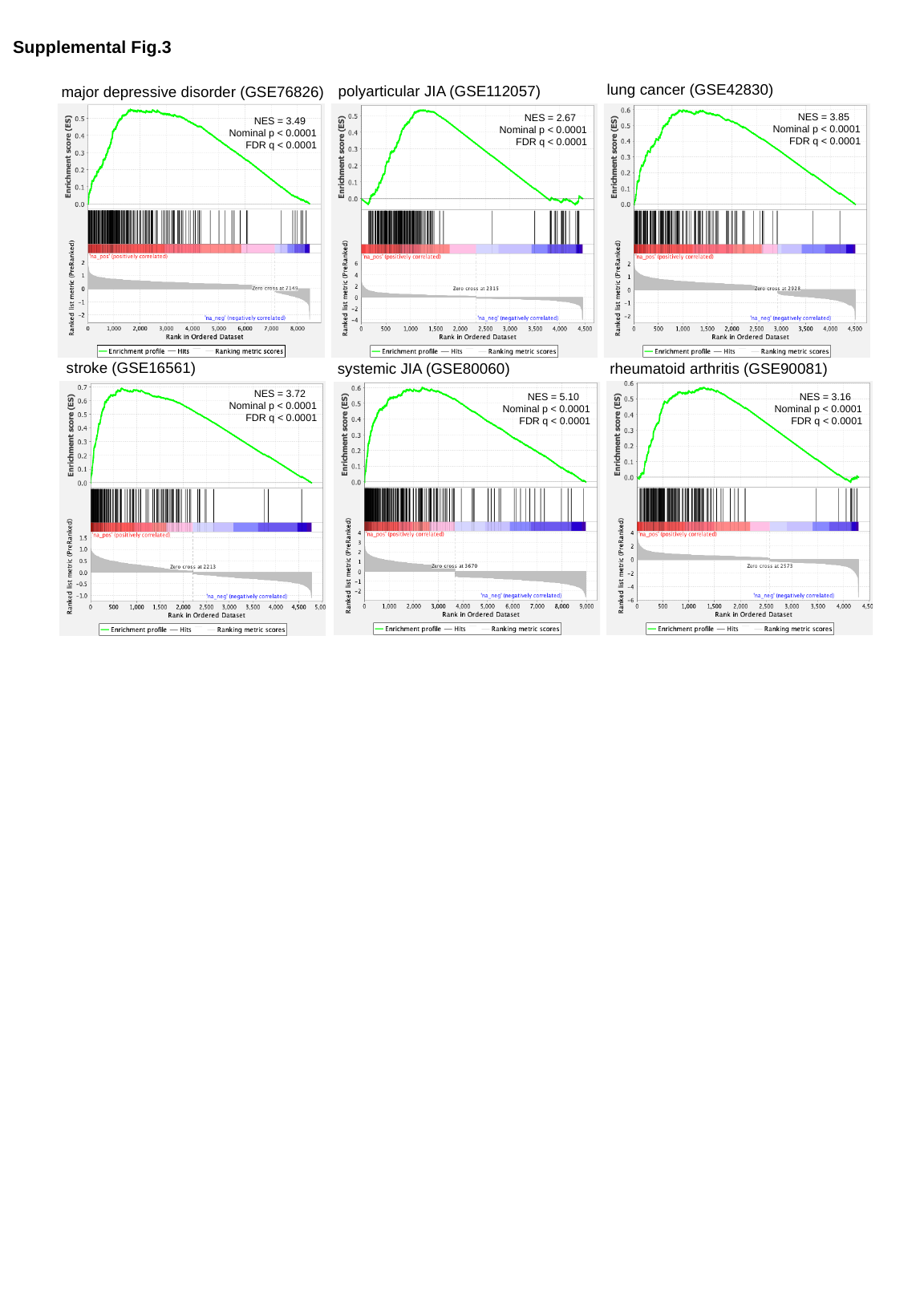

Supplemental Fig.3
lung cancer (GSE42830)
polyarticular JIA (GSE112057)
major depressive disorder (GSE76826)
 NES = 3.85
Nominal p < 0.0001
 FDR q < 0.0001
 NES = 2.67
Nominal p < 0.0001
 FDR q < 0.0001
 NES = 3.49
Nominal p < 0.0001
 FDR q < 0.0001
stroke (GSE16561)
rheumatoid arthritis (GSE90081)
systemic JIA (GSE80060)
 NES = 3.72
Nominal p < 0.0001
 FDR q < 0.0001
 NES = 5.10
Nominal p < 0.0001
 FDR q < 0.0001
 NES = 3.16
Nominal p < 0.0001
 FDR q < 0.0001
