# Supplementary material for: Changes in gene expression in healthcare workers during night shifts: implications for immune response and health risks
Source: J Intensive Care. 2025 Mar 11;13:14. doi: 10.1186/s40560-024-00769-5 (PMC11895378; doi:10.1186/s40560-024-00769-5)
Supplement: Supplementary file 2 — Additional file 2. [file 40560_2024_769_MOESM2_ESM.docx]

| **Supplemental Table 1** Top 5 functional networks in terms of consistency score calculated via regulator effects analysis | | | |
| --- | --- | --- | --- |
| Rank | Predicted regulators | Gene set | Predicted diseases & functions |
| 1 | EP300, FOXO3, HDAC (family), IL17A, IL1B, IL1RN, NFKB (complex), PI3K (complex), SCD, TICAM1, TLR4, TNFSF10, VEGFA | ABCA1, ANGPT2, BCL6, CLEC4E, CXCL8, DDIT4, EGR1, FAS, GADD45G, H2BC4, IFRD1, LDLR, MRC1, NAMPT, NOTCH1, PDK4, PLXDC2, PMAIP1, PTEN, PTGS2, PTPRF, RICTOR, S100A8, SCN9A, SOD2, SRGN, TLR1, TLR2, TLR8 | Activation of astrocytes,  Activation of dendritic cells,  Antimicrobial response,  Development of phagocytes,  Differentiation of antigen presenting cells,  Inflammatory response of endothelial cells,  Learning,  Polarization of macrophages,  Release of lipid,  Sphere formation of tumor cell lines |
| 2 | CD40LG, HDAC (family), HIF1A, IL17A, IL1RN, INTERFERON ALPHA (family), ITPR2, JUNB, NFKB1, NR0B2, PI3K (complex), POR, SCD, SMARCA4, SREBF2, TICAM1, TLR4, TRIM26, VEGFA | ABCA1, ANGPT2, APAF1, BCL6, CLEC4D, CLEC4E, CXCL8, CYP17A1, DDIT4, DICER1, EGR1, ENTPD1, FADS2, FAS, GADD45G, IRS2, LDLR, LY96, MRC1, NAMPT, NOTCH1, PDK4, PMAIP1, PTEN, PTGS2, S100A8, SLC11A1, TLR1, TLR2, TLR6, TLR8, TNFSF10 | Activation of astrocytes,  Activation of dendritic cells,  Antimicrobial response,  Development of antigen presenting cells,  Development of phagocytes,  Differentiation of antigen presenting cells,  Fertility,  Inflammatory response of endothelial cells,  Myelopoiesis of leukocytes,  Recognition of lipid,  Release of lipid |
| 3 | CD40LG, HDAC (family), IFNG, IL1B, INTERFERON ALPHA (family) ,NR0B2, SMARCA4, STAT3, TLR4, TRIM26 | ABCA1, BCL6, CCL23, DICER1, EGR1, FAS, FLT3, GADD45G, IFRD1, LDLR, LY96, NOTCH1, P2RY6, PROK2, PTEN, PTGS2, TLR1, TLR2, TLR6, TLR8, TNFSF10, TNFSF13B | Development of antigen presenting cells,  Differentiation of antigen presenting cells,  Hematopoiesis of phagocytes,  Maturation of leukocytes,  Myelopoiesis of leukocytes,  Recognition of lipid |
| 4 | CYTOKINE (family), HDAC (family), IL17A, NFAT (family), NFKB1, PI3K (complex), SCD, TICAM1, TLR4 | ABCA1, BCL6, CXCL8, DDIT4, DICER1, EGR1, ENTPD1, FAS, FRY, GADD45G, KLF7, LRRK2, MRC1, NAMPT, NOTCH1, PDE3B, PDK4, PTGS2, RAB31, S100A8, SERPINB2, SLC8A1, SOD2, TLR2, TNFSF10 | Activation of astrocytes,  Activation of dendritic cells,  Advanced malignant tumor,  Antimicrobial response,  Development of phagocytes,  Differentiation of antigen presenting cells,  Inflammatory response of endothelial cells,  Invasive cancer,  Polarization of cells,  Release of lipid |
| 5 | CAMP, CIP2A, CREM, CYTOKINE (family), EIF2AK2, GNRH1, IL23 (complex), MAPK3, NFAT (family), NR0B2, TRIM26 | ABCA1, BCL2A1, CLEC4D, CLEC4E, CXCL8, CYP17A1, DICER1,EGR1, EGR2, FAS, GNAQ, IL1RAP, IRS2, KLF5, LDLR, MRC1, NOTCH1, PDE3B, PDK4, PTGS2, RAB31, RGS2, S100A8, SCD, SOD2, TLR1, TLR2, TLR4, TLR6, TLR8, TNFSF13B, USP15, ZDHHC11 | Antimicrobial response,  Cell movement of tumor cell lines,  Cell viability,  Cellular homeostasis,  Development of body trunk,  Immune response of cells,  Invasive cancer,  Leukopoiesis,  Migration of cells,  Organismal death |

| **Supplemental Table 2** Other diseases showing similar expression patterns to those of DEGs after the night shift, as indexed by the dataset match metrics in IPA analysis match | | | |
| --- | --- | --- | --- |
| **Disease (vs. normal)** | **Dataset** | **DM (z-score)** | **DM (p-value)** |
| Major depressive disorder | GSE76826 | 49.4 | 1.09E-104 |
| Polyarticular juvenile idiopathic arthritis | GSE112057 | 46.6 | 3.85E-84 |
| Lung cancer | GSE42830 | 43.6 | 6.72E-64 |
| Stroke | GSE16561 | 43.6 | 9.55E-64 |
| Systemic juvenile idiopathic arthritis | GSE80060 | 43.0 | 3.20E-61 |
| Rheumatoid arthritis | GSE90081 | 42.3 | 1.22E-81 |
| Pulmonary tuberculosis | GSE42832 | 41.4 | 1.01E-54 |
| Myocardial infarction | GSE58294 | 39.5 | 5.41E-53 |
| Systemic sclerosis-associated interstitial lung disease | GSE181228 | 39.3 | 2.28E-61 |
| Glomerulonephritis | GSE222889 | 38.6 | 3.38E-61 |
| Psoriatic arthritis | GSE117769 | 38.4 | 1.19E-64 |
| Systemic lupus erythematosus | E-MTAB-7145 | 37.8 | 7.57E-67 |
| Amyotrophic lateral sclerosis | GSE112676 | 37.3 | 8.76E-40 |
| Pneumonia | GSE42826 | 36.6 | 1.42E-37 |
| Crohn's disease | GSE112057 | 36.6 | 7.74E-57 |
| Cystic fibrosis | GSE38267 | 36.3 | 3.84E-41 |
| Idiopathic pulmonary fibrosis | GSE33566 | 36.2 | 2.43E-36 |
| Intracranial aneurysm | GSE36791 | 35.9 | 7.93E-40 |
| Systemic scleroderma | GSE145120 | 35.5 | 3.42E-39 |
| Ulcerative colitis | GSE119600 | 34.5 | 7.18E-40 |
| *DEGs* differentially expressed genes, *IPA* Ingenuity Pathway Analysis, *DM* dataset match  Top 20 z scores for the strength of correlation: disease name, dataset ID, z score, and p value | | | |
